# Supplementary material for: Dislocation Majorana zero modes in perovskite oxide 2DEG
Source: Sci Rep. 2016 May 3;6:25184. doi: 10.1038/srep25184 (PMC4853714; doi:10.1038/srep25184)
Supplement: Supplementary Information [file srep25184-s1.pdf]

Dislocation Majorana zero modes in perovskite oxide 2DEG: Supplementary Information

Suk Bum Chung,[1,2,*] Cheung Chan,[3,†] and Hong Yao[3,‡]

[1]Center for Correlated Electron Systems, Institute for Basic Science (IBS), Seoul 151-742, Republic of Korea
[2]Department of Physics and Astronomy, Seoul National University, Seoul 151-747, Korea
[3]Institute for Advanced Study, Tsinghua University, Beijing 100084, China

I. RASHBA SPIN-ORBIT COUPLING NEAR VAN HOVE SINGULARITY

The C_{4v} point group symmetry of the (001) perovskite surface 2DEG allows for the spin-momentum coupling in the $d_{xz,yz}$ orbitals from the following nearest-neighbor hopping terms[1,2]:

$$\hat{K}_{RSO} = \alpha_0 \sum_{\mathbf{r}} \Big[c^\dagger_{\mathbf{r}+\hat{\mathbf{e}}_x} is_y\tau_0 c_{\mathbf{r}} - c^\dagger_{\mathbf{r}+\hat{\mathbf{e}}_y} is_x\tau_0 c_{\mathbf{r}} + \text{h.c.}\Big] + \alpha_1 \sum_{\mathbf{r}} \Big[c^\dagger_{\mathbf{r}+\hat{\mathbf{e}}_x} is_x\tau_x c_{\mathbf{r}} - c^\dagger_{\mathbf{r}+\hat{\mathbf{e}}_y} is_y\tau_x c_{\mathbf{r}} + \text{h.c.}\Big] + \alpha_3 \sum_{\mathbf{r}} \Big[c^\dagger_{\mathbf{r}+\hat{\mathbf{e}}_x} is_y\tau_z c_{\mathbf{r}} + c^\dagger_{\mathbf{r}+\hat{\mathbf{e}}_y} is_x\tau_z c_{\mathbf{r}} + \text{h.c.}\Big]. \tag{S1}$$

This raises the question how these terms arise microscopically. Also we can ask whether there is any qualitative effects due to the orbital dependent terms $\alpha_{1,3}$, *i.e.* whether there is any $\sin k_{x,y}$-dependent spin-momentum locking of magnitude in the order of $\alpha_{0,1,3}$ along $k_{x,y} = \pi$.

The starting point for the microscopic physics is that due to the breaking of the inversion symmetry with respect to the xy-plane, there can be spin-independent hopping between $d_{xz,yz}$ orbitals and other d-orbitals:

$$\hat{K}_{inv} = -\gamma_1 \sum_{\mathbf{r}} \sum_{a,a'} (1-\delta_{a,a'}) \Big[c^\dagger_{\mathbf{r}+\hat{\mathbf{e}}_a,a'} s_0 c'_{\mathbf{r}} - c'^\dagger_{\mathbf{r}+\hat{\mathbf{e}}_a} s_0 c_{\mathbf{r},a'} + \text{h.c.}\Big] - \gamma_2 \sum_{\mathbf{r}} \sum_{a,a'} \delta_{a,a'} \Big[c^\dagger_{\mathbf{r}+\hat{\mathbf{e}}_a,a'} s_0 \tilde{c}_{\mathbf{r}} - \tilde{c}^\dagger_{\mathbf{r}+\hat{\mathbf{e}}_a} s_0 c_{\mathbf{r},a'} + \text{h.c.}\Big] - \gamma_3 \sum_{\mathbf{r}} \Big[c^\dagger_{\mathbf{r}+\hat{\mathbf{e}}_x,x} s_0 \tilde{c}'_{\mathbf{r}} - \tilde{c}'^\dagger_{\mathbf{r}+\hat{\mathbf{e}}_x} s_0 c_{\mathbf{r},x} - c^\dagger_{\mathbf{r}+\hat{\mathbf{e}}_y,y} s_0 \tilde{c}'_{\mathbf{r}} + \tilde{c}'^\dagger_{\mathbf{r}+\hat{\mathbf{e}}_x} s_0 c_{\mathbf{r},x} + \text{h.c.}\Big], \tag{S2}$$

where $c', \tilde{c}, \tilde{c}'$ are the annihilation operators for $d_{xy}, d_{z^2}, d_{x^2-y^2}$ orbitals respectively. Note that, in the bulk, any spin-conserving hybridization of this type would not be allowed due the fact that these orbitals are even under the inversion with respect to the xy-plane while the $d_{xz,yz}$ orbitals are odd. But even in the bulk, this hybridization is allowed through the transition metal spin-orbit coupling:

$$\hat{K}'_{aSO} = -\lambda \sum_{\mathbf{r}} \Big[c^\dagger_{\mathbf{r},x}(-is_x)c'_{\mathbf{r}} + c^\dagger_{\mathbf{r},y}(is_y)c'_{\mathbf{r}} + \text{h.c.}\Big] - \sqrt{3}\lambda \sum_{\mathbf{r}} \Big[c^\dagger_{\mathbf{r},x}(-is_y)\tilde{c}_{\mathbf{r}} + c^\dagger_{\mathbf{r},y}(is_x)\tilde{c}_{\mathbf{r}} + \text{h.c.}\Big] - \lambda \sum_{\mathbf{r}} \Big[c^\dagger_{\mathbf{r},x}(is_y)\tilde{c}_{\mathbf{r}} + c^\dagger_{\mathbf{r},y}(is_x)\tilde{c}_{\mathbf{r}} + \text{h.c.}\Big]. \tag{S3}$$

The hybridization of the $d_{xz,yz}$ orbitals with the other d-orbitals through $\hat{K}_{inv} + \hat{K}'_{aSO}$ effectively generates the Rashba-Dresselhaus terms of Eq.(S1) in the subspace of $d_{xz,yz}$ orbitals[1–3]. One relatively simple way to obtain these Rashba-Dresselhaus terms is through the second order degenerate perturbation theory

$$\langle \tilde{a}, \sigma | \hat{\mathcal{K}}_{RSO} | \tilde{a}', \sigma' \rangle = \sum_{b \neq x,y} \frac{\langle \tilde{a}, \sigma | \hat{K}_{inv} | b, \sigma'' \rangle \langle b, \sigma'' | \hat{K}'_{aSO} | \tilde{a}', \sigma' \rangle + \langle \tilde{a}, \sigma | \hat{K}'_{aSO} | b, \sigma'' \rangle \langle b, \sigma'' | \hat{K}_{inv} | \tilde{a}', \sigma' \rangle}{\xi_0 - \xi_b}, \tag{S4}$$

the point here being that we take $\tilde{a}, \tilde{a}'$ to be the band (with spin degeneracy) formed by $d_{xz,yz}$.

One question that arises here is whether the orbital b in Eq.(S4) should include the e_g orbitals as well. Much of the analysis for the Rashba-Dresselhaus term near the Γ point excludes the e_g contributions to Eq.(S4)[1–4], which would be justified in the limit where the e_g orbital energies are much higher than that of the $d_{xz,yz}$ orbitals. Applicability of this limit is qualitatively important for the analysis near the X point, for excluding the e_g contribution effectively gives us

$$\hat{\mathcal{K}}_{RSO} = \frac{4\gamma_1\lambda}{\delta\xi_{xy,yz}} s_y \sin k_x, \tag{S5}$$

where $\delta\xi_{xy,yz}$ is the energy splitting between the d_{xy} and d_{yz} orbitals at the X point, for the d_{yz} orbital (it is straightforward to obtain analogous result for the d_{xz} orbital near the Y point), in which case it would not be justifiable to take the spin-momentum coupling to be $\hat{K}^{(0)}_{RSO}$ of Eq. (3). However, when the inversion symmetry is broken, the energy splitting between the two e_g orbitals actually may be in the same order of magnitude as the crystal field splitting between the t_{2g} and e_g orbitals[5,6]. In such case, the contribution of the lower e_g band (mostly from the d_{z^2} orbital) to Eq.(S4) would give us[7]

$$\hat{\mathcal{K}}_{RSO} \approx \frac{4\gamma_1\lambda}{\delta\xi_{xy,yz}} s_y \sin k_x + \frac{4\sqrt{3}\gamma_2\lambda}{\delta\xi_{z^2,yz}} s_x \sin k_y. \tag{S6}$$

Hence we are justified qualitatively in setting $\hat{K}^{(0)}_{RSO}$ of Eq. (3) to be the spin-momentum coupling in the $d_{xz,yz}$ orbitals[8].

II. ORBITAL MIXING EFFECT NEAR VAN HOVE SINGULARITY

In this Section, we will show that even with a strong orbital hybridization, Eq. (6) still gives us the effective low-energy Hamiltonian at $k_x = \pi$.

As a starting point, let us consider the first-quantized Hamiltonian in the normal state for the d_{xz}/d_{yz} orbitals,

$$\mathcal{H}^{(0)}_{normal} = -(t+t')(\cos k_x + \cos k_y) - \mu - \tau_z(t-t')(\cos k_x - \cos k_y) + \tau_y s_z \lambda + \tau_x 4t'' \sin k_x \sin k_y \tag{S7}$$

(where σ_i's are the orbital Pauli matrices, with the $d_{xz/yz}$ being the eigenstate of σ_3 with the eigenvalue of ± 1), where we have left out the Zeeman and the Rashba terms. When λ is in the same order of magnitude as $t-t'$, as is known for $KTaO_3$, there would be a considerable orbital hybridization even when $k_{x,y} = \pi$. Along $k_x = \pi$, we can relate the band basis and orbital basis through the transformation $U = \exp(i\sigma_1 s_z \beta_{k_y}/2)$ where $\tan\beta_{k_y} = -\lambda/[(t-t')(1+\cos k_y)]$, giving us

$$\begin{aligned} U\, \mathcal{H}^{(0)}_{normal}\Big|_{k_x=\pi} U^\dagger &= (t+t')(1-\cos k_y) - \mu + \tau_z\sqrt{(t-t')^2(1+\cos k_y)^2 + \lambda^2} \\ &\approx (t+t')(1-\cos k_y) - \mu + \tau_z\left[\sqrt{4(t-t')^2+\lambda^2} - \frac{2(t-t')^2(1-\cos k_y)}{\sqrt{4(t-t')^2+\lambda^2}}\right]. \end{aligned} \tag{S8}$$

The low-energy Hamiltonian will then be given by the projection to the lower band, $\sigma_3 = -1$.

We then need to consider how the other terms, which are much smaller in the magnitude, transforms under U. Since it is obvious that the Zeeman term $-s_z h_Z$ and the intra-orbital s-wave pairing $\tau_1|\Delta_s|$ remains invariant, we can mainly focus on the spin-momentum coupled nearest neighbor hopping. From Eq.(S1), we can see that these terms in the first-quantized form comes out to be

$$\mathcal{K}_{SO} = -2\alpha_0(s_x \sin k_y - s_y \sin k_x) + 2\alpha_1\tau_x(s_x \sin k_x - s_y \sin k_y) + 2\alpha_3\tau_z(s_x \sin k_y + s_y \sin k_x). \tag{S9}$$

In the band basis, these terms comes out to be

$$\begin{aligned} U\, \mathcal{K}_{SO}|_{k_x=\pi} U^\dagger &= -2(\alpha_0\cos\beta_{k_y} - \alpha_3\tau_z)s_x \sin k_y - 2\tau_x(\alpha_0 \sin\beta_{k_y} + \alpha_1)s_y \sin k_y \\ &\approx -2\left[\alpha_0\frac{2(t-t')}{\sqrt{4(t-t')^2+\lambda^2}} - \alpha_3\tau_z\right] s_x \sin k_y - 2\tau_x\left[\alpha_0\frac{\lambda}{\sqrt{4(t-t')^2+\lambda^2}} + \alpha_1\right] s_y \sin k_y. \end{aligned} \tag{S10}$$

We can now see that the $\tau_z = -1$ projection of the full BdG Hamiltonian is

$$\begin{aligned} PU\, \mathcal{H}_{BdG}|_{k_x=\pi} U^\dagger P =& \mu_z\left[\left\{(t+t') + \frac{(t-t')^2}{\sqrt{(t-t')^2+\lambda^2/4}}\right\}(1-\cos k_y) - \delta\mu - 2\left\{\alpha_0\frac{(t-t')}{\sqrt{(t-t')^2+\lambda^2/4}} + \alpha_3\right\} s_x \sin k_y\right] \\ & - s_z h_Z + \mu_x|\Delta|, \end{aligned} \tag{S11}$$

where $P = (1-\tau_z)/2$ is the projection operator to the lower band; this is clearly in the same form as Eq. (6).

* sbchung@snu.ac.kr

† phcchan@mail.tsinghua.edu.cn

‡ yaohong@tsinghua.edu.cn

[1] Z. Zhong, A. Tóth, and K. Held, Phys. Rev. B **87**, 161102 (2013).
[2] M. S. Scheurer and J. Schmalian, Nat Commun **6** (2015).
[3] Y. Kim, R. M. Lutchyn, and C. Nayak, Phys. Rev. B **87**, 245121 (2013).
[4] P. Kim, K. T. Kang, G. Go, and J. H. Han, Phys. Rev. B **90**, 205423 (2014).
[5] K. Shanavas and S. Satpathy, Phys. Rev. Lett. **112**, 086802 (2014).
[6] K. V. Shanavas, Z. S. Popović, and S. Satpathy, Phys. Rev. B **90**, 165108 (2014).
[7] M. Kim J. Ihm and S. B. Chung, arXiv:1601.05443.
[8] We note that the inclusion of the d_{z^2} orbital effect near the Γ point insures that the lowest order Rashba term will be linear rather than cubic.
